# Approaches to Intraepithelial Cervical Neoplasia Management in Pregnancy: A Narrative Review

**DOI:** 10.3390/life16050809

**Published:** 2026-05-13

**Authors:** Delia-Maria Bogheanu, Awatif Jaafar Sadeq Al Bayati, Mircea-Octavian Poenaru, Octavian Gabriel Olaru, Gabriel-Petre Gorecki, Andreea Gratiana Boiangiu, Bashar Haj Hamoud, Romina-Marina Sima, Liana Ples

**Affiliations:** 1 Department PhD, IOSUD, “Carol Davila” University of Medicine and Pharmacy, 020021 Bucharest, Romania; delia-maria.bogheanu@drd.umfcd.ro; 2 Department of Obstetrics and Gynecology, “Carol Davila” University of Medicine and Pharmacy, 020021 Bucharest, Romania; mircea.poenaru@umfcd.ro (M.-O.P.); octavian_olaru@umfcd.ro (O.G.O.); andreea.boiangiu@drd.umfcd.ro (A.G.B.); romina.sima@umfcd.ro (R.-M.S.); liana.ples@umfcd.ro (L.P.); 3 “Bucur” Maternity, Saint John Hospital, 012361 Bucharest, Romania; 4 Faculty of Medicine, Titu Maiorescu University, 040441 Bucharest, Romania; gabriel.gorecki@prof.utm.ro; 5 Department of Anesthesia and Intensive Care, CF2 Clinical Hospital, 011464 Bucharest, Romania; 6 Department of Obstetrics and Gynecology, Elias University Emergency Hospital, 011461 Bucharest, Romania; 7 Department for Gynaecology, Obstetrics and Reproductive Medicine, Saarland University Hospital, 66421 Homburg, Germany; bashar.hajhamoud@uks.eu

**Keywords:** cervical dysplasia, cervical screening in pregnancy, colposcopy during pregnancy, postpartum regression, conservative management

## Abstract

This narrative review examines current evidence on the management of cervical intraepithelial neoplasia (CIN) during pregnancy, a clinical scenario requiring a balance between maternal oncologic safety and fetal well-being. A qualitative synthesis was conducted using major databases (PubMed, Google Scholar, Cochrane Library, and ScienceDirect), focusing on studies published between 2019 and 2025, supplemented by key earlier publications. A total of 37 studies and international guidelines were included. Available evidence suggests that pregnancy does not substantially alter the natural history of human papillomavirus infection. Although CIN is generally associated with low progression rates and notable postpartum regression, findings remain heterogeneous and largely based on retrospective data. Colposcopy is essential for excluding invasive disease, while invasive procedures are reserved for suspected malignancy. Most guidelines support conservative management with surveillance during pregnancy and postpartum reassessment. HPV testing offers higher sensitivity, whereas cytology remains widely used due to its specificity. The impact of delivery mode remains inconclusive. Overall, current recommendations rely on limited evidence and should be interpreted with caution. Further prospective studies are needed to refine pregnancy-specific management strategies.

## 1. Introduction

Cervical cancer screening constitutes a vital component of prenatal healthcare. Given that human papillomavirus (HPV) is the primary etiological factor associated with cervical cancer and predominantly affects young women of reproductive age, its implications extend to pregnant populations [1]. The highest prevalence of cervical intraepithelial neoplasia (CIN) 2/3, also referred to as high-grade squamos intaepithelial lesions (HSIL), is observed in women between the ages of 30 to 39 [2]. Research indicates that the incidence of cervical neoplasia complicating pregnancy ranges from 1.4 to 13.1 cases per 100,000 women, with noteworthy regional variations observed [3,4]. Although it is considered that pregnancy does not modify the natural course of HPV infection, and pregnant individuals are thought to exhibit comparable rates of cancer progression to those of non-pregnant individuals [5], management during pregnancy requires careful consideration to avoid both maternal and fetal risks.

A cervical Pap smear examines the cytological characteristics of cervical cells obtained from cervico-vaginal epithelium. This test is effective in diagnosing cervical dysplastic and premalignant lesions, enabling clinicians to initiate appropriate treatment protocols as needed. The management of pregnant women with abnormal cytological results presents a challenge for healthcare providers. It is vital to protect maternal health and eliminate the possibility of invasive lesions, all while making sure that the unborn child is not exposed to avoidable risks. Research has indicated that the incidence of abnormal cervical cytology during pregnancy can be as high as 5% [6]. Despite the fact that changes in the cervix associated with pregnancy can complicate the interpretation of Pap smear results as they can mask a malignant modification [7], Pap smear has traditionally been the standard method for cervical cancer screening [8]. However, recent guidelines prioritize primary HPV testing or co-testing given its superior sensitivity compared to cytology, which remains a complementary and triage tool [5]. Furthermore, pregnancy provides a crucial opportunity to screen women who have not previously received a Pap smear, as they actively seek healthcare services during this period [8,9,10]. The literature indicates that an increasing number of women are experiencing their first pregnancy at later ages, leading to an increased incidence of abnormal cytological findings in pregnant individuals, including atypical glandular cells (AGCs) [11].

Despite growing evidence, the management of cervical dysplasia during pregnancy remains insufficiently standardized. Current recommendations are largely extrapolated from non-pregnant populations and are based on retrospective data, leading to variability in clinical practice and uncertainty regarding optimal management strategies [12].

Therefore, this study places particular emphasis on clinically relevant areas of uncertainty, including the heterogeneity of reported outcomes, the predominance of retrospective evidence, and the potential for unpredictable lesion evolution. By integrating recent data with international guideline recommendations, this review aims to highlight existing gaps and support more individualized clinical decision-making in pregnant patients with CIN.

## 2. Materials and Methods

This study was conducted as a narrative review aiming to examine current evidence regarding screening strategies, diagnostic approaches, natural history, and management of cervical intraepithelial neoplasia during pregnancy. In contrast to systematic reviews, this narrative review was not conducted according to a predefined protocol such as PRISMA, and no formal appraisal of study quality or risk of bias was undertaken. As this is a narrative review, the selection of studies was based on their clinical relevance and contribution to the topic. The aim was to provide a comprehensive synthesis of the available literature, including clinical studies, review articles, and international guidelines, with a focus on identifying key concepts, areas of consensus, and persisting controversies. The literature was explored across several major scientific databases, including PubMed, Google Scholar, the Cochrane Library, and ScienceDirect. The search primarily targeted publications from 2019 to 2025, while earlier studies and landmark papers were also considered where necessary to provide appropriate context. A total of approximately 45 publications were initially identified. After screening titles and abstracts, 37 studies were selected for inclusion based on their relevance and their contribution to key topics such as screening strategies, diagnostic approaches, lesion evolution, and management during pregnancy. In addition, guidelines from major international organizations—including the American Society for Colposcopy and Cervical Pathology (ASCCP), the Society of Obstetricians and Gynaecologists of Canada (SOGC), the Cancer Council Australia, the UK National Health Service (NHS), and the German Society of Gynecology and Obstetrics (DGGG)—were considered to provide a comparative overview of current recommendations.

Given the narrative design of this review, no strict predefined inclusion or exclusion criteria were applied; however, a set of general considerations guided the selection process. Studies were included if they addressed cervical intraepithelial neoplasia in pregnancy and provided clinically relevant data on screening, diagnosis, natural history (progression, persistence or regression) or management strategies. Evidence from retrospective and prospective studies, as well as from meta-analyses, review articles, and clinical guidelines, was considered and is presented descriptively, with differences in study design highlighted where appropriate.

Studies were excluded if they focused exclusively on non-pregnant populations, lacked sufficient clinical detail, were not available in full text (unless the abstract provided relevant information), or were not published in English. Case reports were included selectively when they illustrated clinically relevant or atypical disease evolution. These criteria were applied flexibly, with emphasis on clinical relevance and overall contribution to the understanding of the topic.

The findings were synthesized qualitatively, focusing on screening strategies, diagnostic challenges, patterns of lesion progression and regression, and management approaches during pregnancy. No quantitative analysis or meta-analysis was undertaken. As this study is based exclusively on previously published data, ethical approval was not required.

## 3. Results

Globally, cervical cancer screening has gradually moved from cytology-based approaches toward primary high-risk HPV testing. Although the 2019 ASCCP guidelines established primary HPV testing as the preferred screening modality for non-pregnant women, they also accept co-testing (HPV testing combined with cytology) at 5-year intervals, while cytology alone every 3 years remains an alternative when HPV testing is not available. In pregnancy, however, specific recommendations for HPV-based screening remain limited. As a result, cytology continues to be widely used in clinical practice, with colposcopy serving as the cornerstone of evaluation when abnormalities are detected [5,13].

During pregnancy, the cervix undergoes profound physiological modifications involving both the glandular epithelium and the stromal compartment [14,15]. The endocervical glands frequently display hyperplasia and increased secretory activity, sometimes with nuclear clearing and the potential development of an Arias–Stella reaction, a hormonally induced phenomenon of the endocervical epithelium that may also arise within polyps and mimic adenocarcinoma [14,16]. At the same time, the cervical stroma is subject to collagen degradation through collagenase activity, resulting in edema of the cervical lips, eversion of a substantial portion of the endocervix beyond the external os, and the appearance of an exaggerated ectropion. These changes are often accompanied by marked immature squamous metaplasia and variable degrees of decidualization. In addition, pregnancy-associated cytological alterations may include navicular cells, hyperplastic endocervical cells, and, in rare cases, degenerated trophoblastic cells, all of which may further complicate cytological interpretation. Collectively, these physiological alterations are reflected in Pap smear samples, which can complicate the cytological interpretation of epithelial abnormalities. A large questionnaire-based survey in Poland reported that a substantial proportion of women underwent their first-ever Pap smear during pregnancy, illustrating how gestation often becomes a critical entry point into preventive care [17]. Verma et al. [18] demonstrated in a prospective study that both Pap smear cytology and human papillomavirus deoxyribonucleic acid (HPV-DNA) testing are feasible and safe during pregnancy, with abnormality detection rates comparable to those observed in non-pregnant women. The early second trimester was identified as the optimal period for sample collection due to improved visualization of the transformation zone. Nevertheless, current international guidelines do not provide pregnancy-specific recommendations regarding the preferred screening method, highlighting the gap between emerging evidence and formal guidance. A large cohort study demonstrated that HPV testing—either as a primary method or in combination with liquid-based cytology—offers higher sensitivity than cytology alone for detecting HSIL+ lesions in pregnant women [19]. The results emphasize the importance of genotype-specific evaluation, particularly for HPV16/18 infections, which carried a significantly greater risk of high-grade disease [19]. While the findings support the feasibility and efficiency of incorporating HPV-based strategies into pregnancy-related screening, the lack of pregnancy-specific guideline recommendations remains a limitation. Other literature reviews suggest that HPV-DNA testing may be less useful during pregnancy due to its lower specificity and the high rate of transient positive results, particularly in younger women, where HPV infections are more frequently transient and self-limiting [20,21]. These factors do not appear to alter the overall conservative management strategy, with cytology remaining the preferred method due to its higher specificity for clinically relevant lesions. Moreover, most CIN lesions detected during pregnancy tend to regress postpartum, while progression to invasive cancer remains uncommon [20,21].

### 3.1. Colposcopy in Pregnancy

Colposcopic evaluation during gestation is generally considered safe, yet it presents unique diagnostic challenges due to the extensive hormonal and vascular changes in the cervix. In the early weeks of pregnancy, the cervix appears congested with a characteristic bluish discoloration, known as Chadwick’s sign [22]. Vascular dilatation makes the vessels more prominent and reflects the progesterone-driven decidual transformation [22,23]. In addition, glandular epithelial hyperplasia may present as decidual polyps or nodules, and in some cases is accompanied by epithelial defects appearing as surface erosions [23]. Taken together, these pregnancy-induced modifications can mimic cervical dysplasia or even invasive carcinoma, thereby complicating the accuracy of colposcopic assessment. For this reason, international guidelines consistently recommend that colposcopy in pregnant patients be performed by experienced colposcopists, as insufficient expertise may lead to overestimation of lesion severity and unnecessary interventions or to an increased risk of missed cancers [13]. The 2023 European consensus statement on expert colposcopy emphasizes that the referral criteria for pregnant women with abnormal cervical screening results are identical to those applied to non-pregnant women [24], a recommendation that is likewise endorsed by the 2019 ASCCP guidelines [5]. The principal objective of colposcopy in this setting is not therapeutic but diagnostic—specifically, the exclusion of invasive disease and the provision of reassurance for both clinician and patient. Colposcopic features raising suspicion of invasion include the presence of atypical vascular patterns—such as corkscrew-like or irregularly dilated vessels, endothelial tube formations, and blotchy or disrupted vascular networks—as well as ulcerative changes, friability, and an uneven vascular surface [23]. Endocervical curettage, endometrial biopsy, and treatment without histologic confirmation are contraindicated, while diagnostic excision or repeat biopsy is reserved for cases in which cancer is strongly suspected [5,24]. Safety and role of colposcopy and selective biopsy during pregnancy is echoed in recent clinical reviews: colposcopy is generally safe but technically challenging, and biopsy is appropriate when invasion cannot be excluded, aligning with the conservative approach [13,23,25]. Recent studies have shed light on the correlation between colposcopic impressions and histopathological outcomes in pregnancy. In a 2025 retrospective study involving 125 pregnant women evaluated after abnormal cytology or high-risk HPV positivity, Mi et al. (2025) demonstrated a strong concordance between colposcopic evaluation and biopsy results (κ = 0.82, *p* < 0.001), highlighting the reliability of colposcopy when performed by experienced clinicians with at least 10 years of work experience [26]. Similarly, LeJeune et al. (2025) observed concordance rates of 62.9–68.1% [27], in line with a multicenter study of 69 pregnant women that found a 68.1% agreement and highlighted greater accuracy in the first half of pregnancy (κ = 0.65) [28]. Although some degree of over- and underestimation is still observed, especially in examinations conducted later in pregnancy, the available evidence consistently supports colposcopy as a reliable and safe diagnostic method when carried out by skilled specialists. These results reinforce the importance of performing colposcopy early in gestation under expert supervision, while limiting biopsy to situations where invasive disease cannot otherwise be ruled out.

The approach to managing preneoplastic cervical lesions during pregnancy is a continually evolving and intricate subject. Current research advocates for a conservative strategy, underscoring the necessity of vigilant monitoring. The management of abnormal cytology findings in pregnant women is largely similar to the recommendations established for non-pregnant women.

In order to provide a framework for clinical practice, the following section briefly summarizes the key recommendations from major international guidelines regarding the management of cervical intraepithelial lesions during pregnancy. These recommendations are then contrasted with and complemented by recent findings from the scientific literature.

### 3.2. Management Guidelines Overview

#### 3.2.1. NHS 2024 Updated Cervical Screening and Colposcopy in Pregnancy

Routine cervical screening should be delayed during pregnancy unless there is a history of abnormal results that require earlier attention [29]. If a pregnant person is referred due to an abnormal screening, colposcopy should be done in the late first or early second trimester, unless there is a reason not to. If they had an abnormal colposcopy before becoming pregnant, follow-up should not be delayed.

The main goal of colposcopy during pregnancy is to rule out cancer. Biopsies or treatments are usually postponed until after delivery. However, if there is strong suspicion of cancer, a diagnostic excision may be done during pregnancy, as long as the facility is equipped to manage possible bleeding. If the patient chooses not to have the procedure during pregnancy, they should be reassessed after giving birth.

If low-grade changes (CIN1 or less) are suspected, routine management applies. If high-grade changes (CIN2 or CIN3) are suspected, a repeat colposcopy should be scheduled for the late second trimester or, if that time has passed, three months after delivery. If cancer is suspected, a biopsy is necessary, since a punch biopsy alone may not be enough to rule it out.

#### 3.2.2. British Gynaecological Cancer Society (BGCS) Cervical Cancer Guidelines: Recommendations for Practice

The BGCS Cervical Cancer Guidelines recommend that abnormal cytology during pregnancy be followed by a prompt colposcopy, with the indications for colposcopy being the same for both pregnant and non-pregnant women. The purpose of colposcopy is to exclude invasion, and treatment for pre-invasive conditions should be deferred until after childbirth [30]. If there is suspicion of invasion at colposcopy, then a biopsy adequate for diagnosis should be performed.

#### 3.2.3. ASCCP Guidelines

The ASCCP guidelines emphasize that the management of abnormal cytology in pregnant patients should follow the same clinical action thresholds, surveillance strategies, and colposcopy protocols as those applied to non-pregnant individuals [5]. However, certain procedures that are commonly used in non-pregnant patients are not recommended during pregnancy. For instance, endocervical curettage, endometrial biopsy, and treatment without biopsy are considered unacceptable during pregnancy due to the potential risks they pose to both the mother and the fetus. When a diagnostic excisional procedure or repeat biopsy is considered, it should only be performed if there is a strong suspicion of cancer based on the results of cytology, colposcopy, or histology. This ensures that invasive procedures are reserved for situations where malignancy is a concern and minimizes unnecessary risks during pregnancy.

If CIN 2 or CIN 3 is detected during the first colposcopy examination in pregnancy, follow-up care is recommended. The ASCCP guidelines suggest that these patients undergo regular colposcopy and testing (including cytology and HPV testing, depending on their age) every 12 to 24 weeks throughout pregnancy to help monitor any potential progression of the lesion. However, it is also acceptable to delay colposcopy until after childbirth. While surveillance is important, treatment for histologic HSIL (CIN 2 or CIN 3) during pregnancy is generally not advised unless absolutely necessary.

Colposcopy is recommended no sooner than 4 weeks after delivery during the postpartum period (BII), especially for patients diagnosed with histologic HSIL, in order to allow the cervix to heal.

Furthermore, the 2019 guidelines provide flexibility in managing minor abnormalities, permitting the postponement of colposcopy for women who have previously had negative HPV tests or colposcopy results showing no evidence of CIN grade 2 or worse (CIN 2+) lesions. This approach helps to avoid unnecessary procedures while still ensuring that any significant abnormalities are detected and managed appropriately.

#### 3.2.4. Australian Guidelines

Australian guidelines recommend reviewing cervical screening history during antenatal and postpartum care, offering screening to those due or overdue. Cervical screening is safe during pregnancy when proper techniques are used, avoiding endocervical brushes. For HSIL, conservative management is advised, with colposcopy to rule out invasive cancer, and treatment postponed until after delivery. Postpartum follow-up, including colposcopy and/or HPV testing, should take place 6 weeks to 3 months after delivery. Cervical biopsy is generally unnecessary during pregnancy, unless invasive disease is suspected on colposcopy or predicted by reflex liquid-based cytology sample. The American and Australian Guidelines suggest that women who are high-risk HPV (16/18)-positive in pregnancy should be referred for early colposcopy [31].

#### 3.2.5. Canadian Colposcopy Guidelines

According to the 2023 Canadian Colposcopy Guidelines—developed collaboratively by the Gynecologic Oncology Society of Canada, the Society of Colposcopists of Canada, and the Canadian Partnership Against Cancer—individuals who are pregnant should continue to undergo cervical screening following the established recommendations specific to each province [32]. The criteria for referring individuals to colposcopy remain consistent whether or not the patient is pregnant. In pregnancy, high-risk HPV with normal or low-grade cytology warrants repeat testing 3 months postpartum, while high-grade or glandular cytologic findings require colposcopy within 4 weeks [32]. Guidelines advise against performing endometrial biopsy and endocervical curettage during pregnancy, as these procedures may pose risks to the pregnancy; however, cervical biopsy is recommended when high-grade lesions or cancer are suspected. Also, the guidelines recommend that diagnostic excision for biopsy-confirmed HSIL or Adenocarcinoma In Situ (AIS) may be safely delayed until 8–12 weeks postpartum, as the likelihood of cancer progression during pregnancy is low. Cases of biopsy-confirmed carcinoma or microinvasive carcinoma should be promptly referred to a gynecologic oncologist.

#### 3.2.6. Guidelines of the German Society of Gynecology and Obstetrics and the German Cancer Society

According to the guidelines of the German Society of Gynecology and Obstetrics, the approach to managing preneoplastic cervical lesions in pregnancy aligns closely with the protocols used for non-pregnant women [33]. Procedures such as endocervical curettage and deep endocervical sampling are contraindicated in pregnancy due to potential harm. In cases of CIN 2, CIN 3, or AIS, surgical treatment must be avoided unless invasive cancer cannot be reliably ruled out. These patients should instead undergo close surveillance, with colposcopic evaluations performed every three months. Furthermore, the presence of CIN 2 or 3 should not influence the mode of delivery.

Table 1 offers a synthesized representation of the recommendations reviewed.

### 3.3. Regression, Persistence, and Progression of CIN in Pregnancy as Shown in the Recent Literature

The literature has shown varied rates of progression concerning the natural evolution of CIN during pregnancy. While some research suggests a low progression rate, others indicate a more significant risk, particularly for high-grade lesions. The lowest progression rate reported is between 0% and 0.4%, and it was observed by Origoni et al. in a 2014 literature review on CIN in pregnancy [34]. In their study involving 11,700 pregnant women, Coppolillo et al. (2012) identified 56 cases of CIN 2-3 diagnosed via colposcopically guided biopsy, reporting that 13.3% of these individuals demonstrated progression in the postpartum period [35]. In studies published in the last 5 years regarding natural evolution of CIN in pregnancy, we identified progression rates between 1% and 8.6% [36,37,38,39,40]. (Table 2).

As illustrated in Table 2, the reported outcomes also vary according to lesion grade. For instance, stratified data from Freudenreich et al. [38] demonstrate distinct patterns of regression, persistence, and progression across CIN 1, CIN 2, and CIN 3. Some studies report aggregated outcomes across different CIN grades, such as Suchońska et al. [39]. Investigations published within the last five years have indicated that the incidence of spontaneous regression during the postpartum period among pregnant women with high-grade CIN varies between 20% and 47% [36,37,38,39]. Another literature review indicated that the rate of spontaneous regression falls within the range of 16.7% to 69.3% [42]. This regression may be attributed to the reactivation or restoration of immune system function during the postpartum period [43]. Other studies have shown a relationship between the progression of CIN and the method of delivery [44,45]. The majority of studies have been retrospective. However, in 2013, a prospective clinical study was carried out involving 251 pregnant women who presented with atypical cervical cytology in early pregnancy. Biopsies were performed both during pregnancy and postpartum, revealing lesion progression in 12.3% of cases, while 54.6% showed persistence and 33.1% exhibited regression [46]. The study in question employed an aggressive management approach, which included the use of large loop excisions as part of the treatment strategy.

The natural progression of CIN during pregnancy appears to vary based on the initial grade of the cytologic abnormality. Low-grade lesions tend to exhibit high regression rates and low persistence, whereas high-grade lesions demonstrate lower regression and greater persistence upon postpartum reassessment [47]. Additionally, the risk of progression to microinvasive squamous cell carcinoma is higher for CIN 3 (0–3.2%) compared to CIN 1 (0%) and CIN 2 (0%) [47]. Compared to the natural progression of lesions in non-pregnant individuals, pregnant patients have a higher likelihood of spontaneous lesion regression [43,44]. Although progression is uncommon, the behavior of cervical lesions during pregnancy is not entirely predictable. While many lesions regress postpartum, a substantial proportion persist, and cases of progression—including progression to invasive disease—have been reported, particularly in high-grade lesions. Moreover, emerging evidence suggests that lesion evolution may occasionally be rapid or unexpected, even in patients with previously reassuring findings. Consistent with these data, a 2025 case series from Soroka University Medical Center (*n* = 32) reported no progression to invasive cancer among pregnant patients with biopsy-proven HSIL managed conservatively during gestation; postpartum conization showed 22% regression (15.6% complete; 6.3% to LSIL) and 78.1% persistence, with a 9.4% recurrence rate over a mean 5.7-year follow-up, thereby supporting deferral of excisional treatment until after delivery [48]. Hong D.K. et al. (2019) provided one of the largest retrospective cohorts assessing the clinical outcomes of high-grade CIN diagnosed during pregnancy [49]. Over a ten-year period, 215 pregnant women with biopsy-confirmed CIN2 or CIN3 were followed without excisional procedures during gestation. Postpartum histopathology revealed that while many lesions regressed, a significant proportion persisted, and three cases progressed to invasive cancer. Importantly, persistent high-risk HPV infection—particularly genotypes 16 and 18—emerged as the strongest independent predictor of CIN2+ persistence (OR 5.09, 95% CI 2.15–12.05). In addition, isolated case-based observations have reported rapid CIN progression in non-pregnant populations. For example, Rzymska et al. (2025) described a 32-year-old patient who developed histologically confirmed CIN2 within a short interval following previously normal cytology [50].

### 3.4. Delivery Mode

For individuals with invasive carcinomas, cesarean section is the preferred method of delivery [51,52]. However, for precursor lesions, the effect of delivery mode on regression remains uncertain, as studies present conflicting findings. Some studies favored vaginal delivery over cesarean sections, while others found no significant difference between the two in terms of their impact on the outcome of dysplastic lesions.

Supporters of vaginal delivery argue that the injury and temporary ischemia of the cervix during labor trigger an inflammatory response, which promotes repair and cervical remodeling through the buildup of inflammatory cells [42]. Additionally, the passage of the fetus through the birth canal and cervical ripening may result in the loss of dysplastic foci, contributing to CIN regression [42].

A comprehensive analysis of eight retrospective studies involving 813 patients whose premalignant lesions were evaluated cytologically, of whom 685 delivered via the vaginal route, and 233 patients whose squamous intraepithelial lesions were evaluated histologically, of whom 162 delivered vaginally, revealed no significant differences in the regression, persistence, or progression of premalignant cervical lesions between vaginal and cesarean deliveries, indicating that the mode of delivery does not influence the natural course of these lesions [45]. Another retrospective study, including 219 women, of whom 141 delivered vaginally and 51 had a cesarean section, assessed antepartum and postpartum outcomes. The study recvealed that the regression rates for cervical lesions were similar between the two groups, with 36% for vaginal deliveries and 47% for cesarean sections, suggesting that the mode of delivery does not significantly affect the outcome of CIN [45]. Other studies also suggest that there is no difference in outcomes between cesarean section and vaginal delivery in cases of CIN lesions, regardless of their grade [38,39,47,53]. On the contrary, a retrospective study conducted by Stuebs F.A. including 655 patients found a higher regression rate after vaginal delivery [41]. In another research article, Stuebs F.A. et al. reported that the rate of regression was nearly twice as high with vaginal delivery (27.4%) compared to cesarean section (15.2%), while the progression rate was lower in women who delivered vaginally (2.7%) than in those who underwent cesarean section (6.5%) [37].

Also, in their retrospective study of 64 pregnant women with abnormal cytologic findings, Chung et al. found that 45 of those women (70.3%) delivered vaginally and 19 women (29.7%) delivered via cesarean section. They observed a postpartum regression rate of 92.9% in the women who delivered vaginally (39 women) and 63.2% in those who underwent cesarean section (12 women) [54].

The influence of the delivery method on the outcome of cervical dysplastic lesions remains a subject of ongoing debate. Additional research involving larger sample sizes and diverse geographical locations is needed to gain a clearer understanding of this issue.

### 3.5. Psychological Burden

A dimension largely overlooked in guidelines is the psychological burden associated with a diagnosis of CIN during pregnancy. Recent evidence demonstrates significant levels of anxiety and reduced quality of life among affected women, underscoring the need to integrate psychosocial support into management strategies. Recent evidence shows that a diagnosis of CIN is frequently accompanied by clinically relevant psychological distress in non-pregnant populations. Scherer-Quenzer et al. reported that 57% of women with cervical dysplasia experienced clinically significant distress, particularly younger and less-educated patients [55]. Although progression to invasive cancer during pregnancy is rare, Suchońska et al. noted that the uncertainty and postponement of treatment may heighten maternal anxiety [39]. However, no studies to date have specifically assessed the psychological impact of CIN diagnosis during pregnancy, leaving this dimension largely underexplored.

## 4. Discussion

The management of CIN during pregnancy represents a complex clinical scenario that requires balancing maternal oncologic safety with fetal well-being. While the available literature and international guidelines consistently support a predominantly conservative approach [5,30,31,32,33], this strategy should be interpreted within the context of important limitations and areas of uncertainty.

The profound physiological alterations associated with pregnancy, such as increased cervical vascularity, glandular hyperplasia, and stromal remodeling, pose significant challenges to both cytological and colposcopic evaluation [14,15,23]. By modifying the appearance of cervical tissue, these changes may obscure underlying pathology and contribute to diagnostic uncertainty, including both under- and overestimation of lesion severity. Therefore, although colposcopy continues to represent the cornerstone of assessment [5,24], its diagnostic reliability is contingent upon examiner expertise and is further constrained by the inability to perform certain procedures, including endocervical curettage, in the pregnant population.

Another area of ongoing debate concerns the role of HPV-based screening in pregnant populations. Although HPV testing demonstrates higher sensitivity for the detection of high-grade lesions [19], its lower specificity and the high prevalence of transient infections during pregnancy [20,21] may result in increased false-positive rates with limited impact on the overall conservative management strategy. The absence of pregnancy-specific screening guidelines highlights a gap between emerging evidence and clinical practice, underscoring the need for further research to define optimal strategies in this setting.

The natural history of CIN during pregnancy is generally considered favorable; however, this interpretation warrants cautious consideration. Although postpartum regression rates are substantial [36,37,38,39,42], persistence remains common—particularly in high-grade lesions—and may exceed 50–70% in some cohorts [37,41]. Furthermore, while progression to invasive carcinoma is rare [34], it is not negligible, and cases of malignant transformation have been reported [49].

Considerable variability across studies may be attributed to differences in study design, patient populations, diagnostic methods, and duration of follow-up [36,37,38,39,40]. In addition, the inclusion of heterogeneous lesion grades and the use of aggregated data in some analyses [39] limit direct comparison between findings. Reported regression rates may also be influenced by the timing of postpartum evaluation, as regression can continue beyond early follow-up intervals. These factors, together with the predominance of retrospective studies, may introduce bias and affect the accuracy of reported outcomes. Moreover, variations in diagnostic or management strategies across studies may further influence the reported rates of progression, persistence, and regression. Importantly, the evolution of CIN is not always linear, and instances of rapid or unpredictable progression have been described [50], challenging the assumption that all lesions follow a slow and benign course. Therefore, the apparent reassurance provided by low progression rates should be interpreted in the context of potential atypical disease behavior and the methodological limitations of the available evidence. Additionally, much of the existing guidance is extrapolated from non-pregnant populations [5], potentially overlooking pregnancy-specific biological and immunological factors that may influence lesion behavior. In this context, the cervicovaginal microbiota may represent an additional factor influencing HPV persistence and cervical disease progression. A Lactobacillus-dominated microbiota is generally associated with a protective environment, whereas dysbiosis has been linked to increased HPV persistence and local inflammation [56]. Although current evidence is largely derived from non-pregnant populations, pregnancy-related immunological and hormonal changes may further modulate this interaction.

Most international recommendations converge on a conservative management strategy during pregnancy, with the primary objective being the exclusion of invasive disease while minimizing fetal risk [5,30,31,32,33]. At the same time, the emphasis on caution, particularly the avoidance of invasive procedures and the preference for surveillance, inevitably influences the diagnostic process. While this approach is appropriate in the context of fetal safety, it may also result in a less comprehensive assessment of lesion severity during pregnancy. As a consequence, certain lesions may not be fully characterized until the postpartum period. This highlights the importance of careful clinical judgment, appropriate patient selection, and structured postpartum follow-up to ensure that clinically significant disease is not overlooked.

These considerations should also be interpreted in light of the limitations of the present review. As a narrative review, it does not follow a standardized systematic methodology and does not include a formal assessment of study quality or risk of bias. In addition, the selection of studies may involve a degree of subjectivity, and the findings are derived from heterogeneous evidence with varying study designs and follow-up durations. Therefore, the conclusions presented should be interpreted with appropriate caution.

With regard to obstetric management, the influence of delivery mode on CIN outcomes remains controversial. Although most studies and meta-analyses suggest no significant impact [38,39,45,47], conflicting data indicate a possible association between vaginal delivery and higher regression rates [37,42]. This inconsistency likely reflects differences in study design, sample size, and lesion classification, and currently does not support a change in clinical practice. As such, mode of delivery should continue to be determined based on obstetric indications rather than the presence of CIN alone.

An additional, often underrecognized dimension is the psychological burden associated with a diagnosis of CIN during pregnancy. Anxiety, uncertainty, and the postponement of treatment may significantly affect maternal well-being [55], yet this aspect remains insufficiently explored in the literature. Integrating psychosocial support and clear patient communication into clinical care may therefore represent an important component of management.

## 5. Conclusions

Overall, while conservative management remains the standard of care, it should not be applied uniformly without consideration of individual risk factors. Careful patient selection, close surveillance, and structured postpartum follow-up are essential to ensure optimal outcomes. Future research should prioritize well-designed prospective studies focused specifically on pregnant populations in order to address existing gaps and refine evidence-based recommendations.

## Figures and Tables

**Table 1:** Guidelines summary.

Guideline	Routine Screening	Abnormal Results	Biopsy	Management of High-Grade Lesion	Postpartum Follow-Up
NHS (UK) [29]	Delayed unless there is a history of abnormal results	Low-grade changes routine management applies	If cancer is suspected	Repeat colposcopy in late second trimester or 3 months after delivery	Colposcopy 3 months after delivery
BGCS (UK) [30]	Same as non-pregnant patients; colposcopy if abnormal cytology	Colposcopy indicated to exclude invasion	Not routinely performed during pregnancy	Treatment deferred until after delivery	Colposcopy after delivery
ASCCP (USA) [5]	Same thresholds and surveillance as non-pregnant patients	Colposcopy required	Cervical biopsy only if cancer suspected	HSIL diagnosed at the first colposcopy during pregnancy; monitor with colposcopy/cytology every 12–24 weeks; deferring colposcopy to the postpartum period is acceptable; AISreferral to a gynecologic oncologist	Colposcopy no earlier than 4 weeks postpartum
Cancer Council Australia [31]	Screening recommended if due	Colposcopy if HSIL or HPV 16/18 positive	Avoided unless invasion suspected	Conservative management; defer treatment	Colposcopy/HPV testing no less than 6 weeks, preferably at 3 months postpartum
SOGC (Canada) [32]	Follow screening protocols, same protocols as for non-pregnant patients	Risk-based indications for colposcopy; the same regardless of pregnancy	Biopsy if HSIL or carcinoma suspected	Colposcopy within 4 weeks for high-grade/glandular results Delay excision for HSIL/AIS unless carcinoma suspected	Excisional procedures for biopsy-proven HSIL or AIS delayed until 8–12 weeks postpartum ASC-US or LSIL-HPV-based screening repeated 3 months postpartum
DGGG and the DKG (Germany) [33]	Same protocols as for non-pregnant patients	Excluded high-grade dysplasia and cancer = no further colposcopy and/or cytological investigations in pregnancy	Biopsy if needed	Colposcopy every 3 months if CIN 2/3/ACIS suspected Avoid surgery unless cancer cannot be ruled out	Continue surveillance postpartum; delivery method unchanged

LSIL—Low-Grade Squamous Intraepithelial Lesion; ASC-US—Atypical Squamous Cells of Undetermined Significance; HSIL—High-Grade Squamous Intraepithelial Lesion; AIS—Adenocarcinoma In Situ.

**Table 2:** Regression, persistence and progression of CIN during pregnancy.

Study	*N*	CIN	Study Design	Regression%	Persistence%	Progression%
Freudenreich, R. et al. [38] (2022)	142	CIN 1 CIN 2 CIN 3	Retrospective	37.1 35.3 15.4	54.3 23.5 64.1	8.6 41.2 2.6
Grimm, D. (2020) [40]	139	CIN 2–3	Retrospective	37.3	61.7	1
Suchońska et al. 2020 [39]	53	CIN 1–2+	Prospective	47.1	50	2.9
Stuebs, F.A. et al. 2023 [37]	154	CIN 3	Retrospective	20	76.1	3.2
Chen, C. et al. (2021) [36]	832 from 10 studies	CIN 2–3	Meta-analysis	40	59	1
Stuebs, F.A. 2023 [41]	655	CIN 3		21.9	80	4.1
		CIN 2		31.3	37.5	-

## Data Availability

Not applicable.

## Abbreviations

The following abbreviations are used in this manuscript:

AGC	Atypical glandular cells
AIS	Adenocarcinoma in situ
ASC-US	Atypical squamous cells of undetermined significance
ASCCP	American Society for Colposcopy and Cervical Pathology
BGCS	British Gynaecological Cancer Society
CIN	Cervical intraepithelial neoplasia
DGGG	German Society of Gynecology and Obstetrics
DKG	German Cancer Society
HPV	Human papillomavirus
HSIL	High-grade squamous intraepithelial lesion
LSIL	Low-grade squamous intraepithelial lesion
NHS	National Health Service
SOGC	Society of Obstetricians and Gynaecologists of Canada
